# Insulin‐stimulated Rac1‐GTP binding is not impaired by palmitate treatment in L6 myotubes

**DOI:** 10.14814/phy2.13956

**Published:** 2018-12-27

**Authors:** Harrison D. Stierwalt, Sarah E. Ehrlicher, Bryan C. Bergman, Matthew M. Robinson, Sean A. Newsom

**Affiliations:** ^1^ School of Biological and Population Health Sciences College of Public Health and Human Sciences Oregon State University Corvallis Oregon; ^2^ Division of Endocrinology, Metabolism and Diabetes School of Medicine University of Colorado Denver Denver Colorado

**Keywords:** Insulin signaling, PAK, Rac1, skeletal muscle

## Abstract

Ras‐related C3 botulinum toxin substrate 1 (Rac1) is required for normal insulin‐stimulated glucose transport in skeletal muscle and evidence indicates Rac1 may be negatively regulated by lipids. We investigated if insulin‐stimulated activation of Rac1 (i.e., Rac1‐GTP binding) is impaired by accumulation of diacylglycerols (DAG) and ceramides in cultured muscle cells. Treating L6 myotubes with 100 nmol/L insulin resulted in increased Rac1‐GTP binding that was rapid (occurring within 2 min), relatively modest (+38 ± 19% vs. basal, *P* < 0.001), and short‐lived, returning to near‐basal levels within 15 min of continuous treatment. Incubating L6 myotubes overnight in 500 *μ*mol/L palmitate increased the accumulation of DAG and ceramides (*P* < 0.05 vs. no fatty acid control). Despite significant accumulation of lipids, insulin‐stimulated Rac1‐GTP binding was not impaired during palmitate treatment (*P* = 0.39 vs. no fatty acid control). Nevertheless, phosphorylation of Rac1 effector protein p21‐activated kinase (PAK) was attenuated in response to palmitate treatment (*P* = 0.02 vs. no fatty acid control). Palmitate treatment also increased inhibitory phosphorylation of insulin receptor substrate‐1 and attenuated insulin‐stimulated phosphorylation of Akt at both Thr308 and Ser473 (all *P* < 0.05 vs. no fatty acid control). Such signaling impairments resulted in near complete inhibition of insulin‐stimulated translocation of glucose transporter protein 4 (GLUT4; *P *= 0.10 vs. basal during palmitate treatment). In summary, our finding suggests that Rac1 may not undergo negative regulation by DAG or ceramides. We instead provide evidence that attenuated PAK phosphorylation and impaired GLUT4 translocation during palmitate‐induced insulin resistance can occur independent of defects in insulin‐stimulated Rac1‐GTP binding.

## Introduction

Obesity is strongly linked with the development of impaired insulin‐stimulated glucose uptake in skeletal muscle, which is a risk factor for type 2 diabetes (DeFronzo 1988). Skeletal muscle accounts for up to 80% of glucose uptake in the postprandial state (DeFronzo and Tripathy 2009) and thus it is a significant regulator of systemic glucose metabolism. It is therefore critical to understand mechanisms underlying skeletal muscle insulin resistance with obesity, including the role for lipids as regulators of skeletal muscle glucose uptake (Bajaj et al. 2005; Frangioudakis and Cooney 2008; Schenk et al. 2009).

Ras‐related C3 botulinum toxin substrate 1 (Rac1) is a Rho‐family GTPase that is required for normal insulin‐stimulated glucose transport in skeletal muscle (Chiu et al. 2011; Sylow et al. 2013, 2014). GTP‐bound Rac1 induces reorganization of cortical actin‐filaments near the sarcolemmal membrane which then facilitate the translocation of glucose transporter vesicles to the cell surface (JeBailey et al. 2007; Ueda et al. 2008, 2010). Insulin‐stimulated “release” of GLUT4 vesicles from intracellular compartments requires activation of protein kinase B (Akt) (Eguez et al. 2005; Larance et al. 2005). Evidence indicates both Rac1 and Akt are activated downstream of phosphatidylinositol 3‐kinase (PI3K) (Sylow et al. 2014). Activation of both Akt and Rac1 is required for normal insulin‐stimulated glucose transport, but these pathways may otherwise be independent (Sylow et al. 2014). For example, overexpression of a dominant negative Akt impairs insulin‐stimulated GLUT4 translocation without affecting Rac1 signaling or actin filament reorganization (Sylow et al. 2014). Similarly, inducible knockout of skeletal muscle Rac1 attenuates insulin‐stimulated GLUT4 translocation without impairing Akt phosphorylation (Sylow et al. 2014; Raun et al. 2018). Taken together, Rac1 has emerged as a critical regulator of skeletal muscle glucose uptake.

Skeletal muscle Rac1 signaling was recently shown to be impaired in models of insulin resistance characterized by excess availability of lipids, including obese humans, high fat‐fed mice and during acute infusion of lipids (i.e., intralipid) (Sylow et al. 2013, 2014). These studies report Rac1 signaling using phosphorylation of a downstream target, p21 activated kinase (PAK). Direct measures of Rac1 activation (i.e., GTP binding) are therefore needed to confirm impairment to Rac1 in skeletal muscle insulin resistance. Furthermore, regulatory mechanisms responsible for impaired activation of Rac1 signaling during lipid‐induced skeletal muscle insulin resistance remain unclear. An intriguing possibility is that Rac1 may be negatively regulated by the accumulation of intramyocellular lipids. Both diacylglycerols (DAG) and ceramides are implicated in the development of obesity‐related insulin resistance (Powell et al. 2004; Ritter et al. 2015; Chung et al. 2017; Perreault et al. 2018), and can alter protein function via allosteric binding of C1 domains (Yin et al. 2009; Szendroedi et al. 2014). C1 domains are common among proteins that regulate Rac1 and other Rho‐family GTPases (Colón‐González and Kazanietz 2006) and may serve as a mechanism for DAG and ceramides to regulate Rac1. Indeed, DAG is known to negatively regulate Rac1 in nonskeletal muscle tissues (Wang and Kazanietz 2006; Sosa et al. 2009). However, to what extent Rac1‐GTP binding may be negatively regulated by accumulation of lipids in skeletal muscle cells remains unclear.

The purpose of this study was to identify the role for lipids as negative regulators of Rac1 in muscle cells. We hypothesized that accumulation of DAG and ceramides in L6 muscle cells would impair insulin‐stimulated Rac1‐GTP binding, leading to attenuated activation of PAK and decreased GLUT4 translocation. In contrast to our hypothesis, insulin‐stimulated Rac1‐GTP binding was not impaired in palmitate‐treated cells despite significant accumulation of DAG and ceramides. Nevertheless, PAK phosphorylation and GLUT4 translocation were attenuated during treatment with palmitate, suggesting that these impairments may occur independent of defects in insulin‐stimulated Rac1‐GTP binding.

## Methods

### Cell culture

L6 myoblasts (CRL‐1458; American Type Culture Collection) were grown in high glucose (4.5 g/L) Dulbecco's modified Eagle's medium (DMEM) supplemented with 10% fetal bovine serum (FBS) and 1% antibiotic/antimycotic (AbAm) in a 5% CO_2_ humidified atmosphere at 37°C. Cells at ~80–90% confluence were differentiated into myotubes by switching to low glucose (1 g/L) DMEM supplemented with 2% horse serum and 1% AbAm for 5–7 days. Fatty acid treatments were prepared in ethanol and added to 37°C differentiation media supplemented with 2% bovine serum albumin (BSA) and 1 mmol/L carnitine, at a final concentration of 500 *μ*mol/L with 0.5% ethanol. A no fatty acid control treatment consisted of differentiation media supplemented with 2% BSA, 1 mmol/L carnitine, and 0.5% ethanol. Myotubes were incubated for 22 h with palmitate (PALM), oleate (OLEA), or no fatty acid control (CON) treatments, with the final 4 h of incubation involving serum starvation (i.e., treatment media devoid of horse serum). Insulin treatment consisted of 100 nmol/L insulin for durations ranging from 2 to 15 min, as described for individual experiments. CN04, a pharmacological activator of Rac1 (Cytoskeleton Inc.), was used according to the manufacturer recommendations at a concentration of 1 *μ*g/mL for 4 h. Culture media and supplements were purchased from Gibco and Millipore‐Sigma. Details regarding the number of experiments for each analytical method are provided below.

### Lipid analysis

L6 myotubes grown in 10 cm plates were harvested in ddH_2_O and transferred to a glass screw cap tube with methanol (MeOH), methyl tert‐butyl ether (MTBE), and internal standards. Samples were then vortexed, rotated for 5 min at room temperature, and centrifuged at 2500*g* for 5 min to separate phases. The upper phase containing lipids was transferred to new glass culture tubes. The lower phase was repeat‐extracted with an additional MTBE, MeOH, and acidified H_2_O. The combined extracts were dried under nitrogen gas and low heat (~37°C). The total lipid extract was transferred to autosampler vials using 2:1 chloroform:methanol, then re‐dried under nitrogen gas. Lipids were resuspended in 95:5:0.1 hexane:dichloromethane:actic acid for analysis. DAG and ceramide species were analyzed using an Agilent 1100 high performance liquid chromatographer connected to a Sciex API2000 triple quadrupole mass spectrometer. The mass spectrometer was used in multiple reaction monitoring mode. 1,3‐ and 1,2‐DAG isomers were separated chromatographically using a Hilic 2.1 *μ*mol/L, 2.1 × 100 mm column with a two‐stage gradient of mobile phase from 2%B to 64%B (A = isooctane; B = 705:20:75, isooctane:acetonitrile:isopropanol). Post column addition of 30 mmol/L ammonium acetate in 95:5 isopropyl alcohol:water was used to promote formation of ammonium adducts of the neutral lipid species in the electrospray ion source of the mass spectrometer. Standard curves were generated with reference standards combined with the same quantity of internal standard cocktail added to samples upon extraction. The concentration of each molecular species in the samples was determined by comparing area ratio obtained by dividing the peak area of analyte by the peak area of its internal standard to standard curves. Lipid species were quantified using MultiQuant software (Sciex). Lipid analysis reflect two independent experiments each consisting of *n* = 3–4 for a total sample size of *n* = 7 for all conditions.

### Rac1 activation

Rac1‐GTP binding was measured via immunosorbent assay according to the manufacturer recommendations (BK128, Cytoskeleton Inc.). Myotubes grown in 6‐well plates were harvested in lysis buffer plus protease inhibitors. Cell lysates were centrifuged for 1 min at 10,000*g* and flash frozen in liquid nitrogen. Sample protein concentrations were equalized and loaded into wells coated with Rac1‐GTP binding domain. GTP‐bound Rac1 was determined following a colorimetric reaction using antibodies toward Rac1 linked with horseradish peroxidase activity. Rac1 activation analyses during basal, 2, 5, and 10 min of insulin stimulation reflect three independent experiments each consisting of *n* = 3 for a total sample size of *n* = 9. Insulin‐stimulated Rac1 activation at 15 min and during CN04 treatment were each assessed during one experiment with a sample size of *n* = 3.

### GLUT4 translocation

L6 myotubes with stable transfection of *myc*‐tagged GLUT4 (L6‐GLUT4*myc*; Kerafast Inc.) were used to determine insulin‐stimulated GLUT4 translocation as previously described (Wang et al. 1998). Briefly, L6‐GLUT4*myc* myoblasts were grown in minimum essential medium alpha (*α*MEM) supplemented with 10% FBS and 1% AbAm and differentiated by switching to *α*MEM supplemented with 2% FBS and 1% AbAm. Fatty acid and control treatments were prepared and executed as described above (see Cell Culture) using *α*MEM media. Latrunculin B (428020, Millipore‐Sigma), a pharmacological inhibitor of actin filament remodeling, was prepared in DMSO and provided at a final concentration of 5 *μ*mol/L for 1 h (Sylow et al. 2014). Myotubes were stimulated with 100 nmol/L insulin for 20 min. For detection of cell‐surface GLUT4*myc*, myotubes were rinsed with phosphate buffered saline (PBS), fixed in 4% paraformaldehyde in PBS, blocked with 5% goat serum in PBS, and incubated with anti‐c‐myc polyclonal antibody (C3956, Millipore‐Sigma). Myotubes were extensively rinsed with PBS before incubation in horseradish peroxidase‐conjugated goat anti‐rabbit secondary antibody (111‐035‐114, Jackson ImmunoResearch). Myotubes were rinsed with PBS before o‐phenylenediamine reagent (P5412, Millipore‐Sigma) was added to each well. The colorimetric reaction was stopped by the addition of 3M HCl. Supernatant was collected and the optical absorbance was measured at 492 nm. GLUT4 translocation analyses reflect three independent experiments each consisting of *n* = 3 for a total sample size of *n* = 9 for fatty acid and no fatty acid control conditions. Latrunculin B treatments included one experiment for basal conditions and two independent experiments during insulin‐stimulated conditions, each consisting of *n* = 3 for a total sample size of *n* = 3 and *n* = 6, respectively.

### Western blotting

Western blot analysis was performed on cell lysates as previously described (Newsom et al. 2017). L6 myotubes grown in 6‐well plates were harvested in lysis buffer plus protease inhibitors and centrifuged at 10,000*g* for 10 min at 4°C with the supernatant stored at −80°C until analysis. Approximately 30 *μ*g protein was separated on bis–tris gels then transferred to nitrocellulose membranes. Each gel was loaded with the same internal control sample in two lanes, the average density of both lanes was used to normalize band density between gels. Ponceau staining of membranes was used to verify equal loading and transfer of protein. Membranes were blocked in 5% bovine serum albumin in tris‐buffered saline with tween (TBST) and incubated in primary antibodies at 4°C. Membranes were washed in TBST and incubated in secondary antibody diluted in blocking buffer at room temperature. Images were generated using infrared detection (LI‐COR Odyssey). Primary antibodies used included Rac1 (ARC03; Cytoskeleton Inc.) and Akt (2920), pAkt Ser473 (9271), pAkt Thr308 (9275), pPAK1/2 Thr423/Thr402 (2601), PAK1 (2602), pIRS1 Ser1101 (2385), IRS1 (3194), pPDK1 Ser241 (3438), and PDK1 (3062) from Cell Signaling Technology. Secondary antibodies used included anti‐rabbit‐700 (926‐68071) and anti‐mouse‐800 (926‐32212) from LI‐COR. Insulin signaling measures via western blotting reflect three independent experiments each consisting of *n* = 3 for a total sample size of *n* = 9 for all conditions.

### Statistical analysis

Initial characterization of Rac1‐GTP binding was analyzed by one‐way ANOVA, with Dunnett's posthoc analysis comparing insulin‐stimulated time points and CN04 treatment to basal. Intracellular lipid abundance was analyzed by one‐way ANOVA, with Dunnett's posthoc analysis comparing fatty acid treatments to the no fatty acid control condition. Insulin signaling outcomes were analyzed by two‐way *time* *× treatment* ANOVA. A main effect of insulin was evaluated by comparing 2, 5, and 10 min of insulin stimulation with basal using Dunnett's analysis. Main effects of fatty acid treatments were evaluated by comparing palmitate and oleate with the no fatty acid control condition using Dunnett's analysis. Cell‐surface GLUT4*myc* during basal and insulin‐stimulated conditions was analyzed via unpaired two‐tailed students *t*‐test. Statistical significance was set as *P *<* *0.05. Statistical analysis was performed using Prism version 6 (GraphPad Software). In accordance with published recommendations (Curran‐Everett 2008), data are presented as mean + SD.

## Results

### Insulin‐stimulated Rac1 activation is short‐lived in L6 myotubes

**Figure 1:**
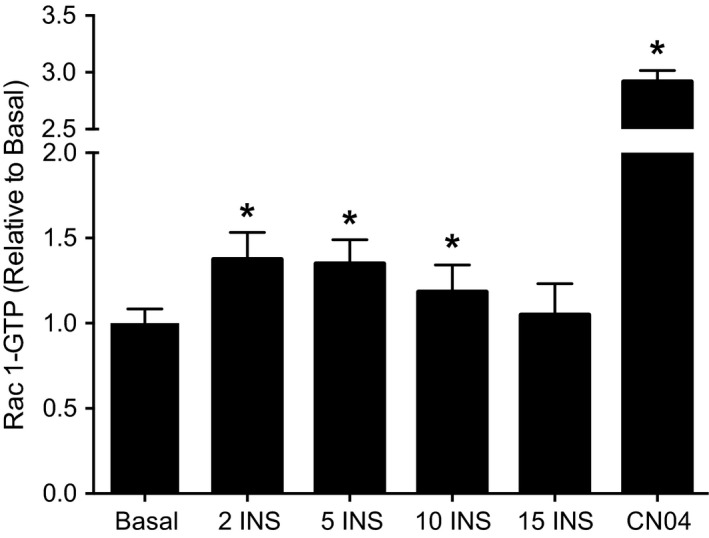
Insulin‐stimulated Rac1‐GTP binding in L6 myotubes. Rac1‐GTP binding was assessed via immunosorbent assay in L6 myotubes under basal serum‐free conditions and 100 nmol/L insulin (INS) for 2, 5, 10, and 15 min. For comparison, L6 myotubes were incubated for 4 h with CN04, a pharmacological treatment to maximize Rac1‐GTP binding (Lerm et al. 2002). Rac1‐GTP binding was analyzed by one‐way ANOVA with Dunnett's posthoc analysis comparing each time point and CN04 to basal. Rac1‐GTP binding during basal, 2, 5, and 10 min of insulin stimulation reflects three independent experiments each consisting of *n* = 3 for a total sample size of *n* = 9. Insulin‐stimulated Rac1‐GTP binding at 15 min and during CN04 treatment each reflect 1 experiment with a sample size of *n* = 3. Data are presented as mean + SD. *P < 0.05 versus Basal.

To address our primary aim, we first sought to characterize insulin‐stimulated Rac1‐GTP binding in L6 myotubes. Incubating myotubes in 100 nmol/L insulin resulted in a rapid increase in GTP binding, occurring within 2 min; yet the increase was transient, returning to near‐basal levels within 15 min of continuous insulin stimulation (Fig. 1). Further, the insulin‐stimulated increase in Rac1‐GTP binding was relatively modest (+38 ± 19% vs. basal) compared with pharmacological activation of Rac1 using CN04 (+191 ± 11% vs. basal, Fig. 1). Together these data indicate insulin‐stimulated Rac1 activation in L6 myotubes is rapid, short‐lived, and relatively modest.

### DAG and ceramides accumulate during palmitate treatment in L6 myotubes

**Figure 2:**
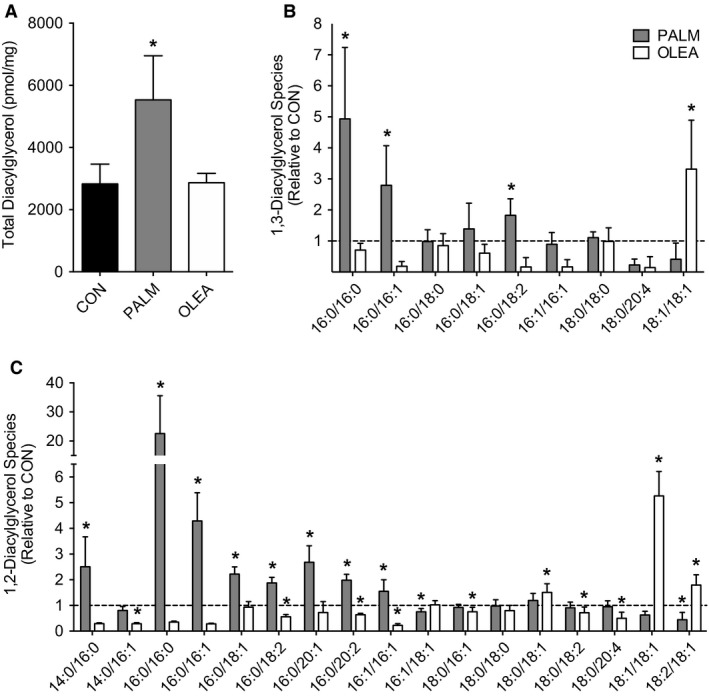
Diacylglycerol abundance following overnight fatty acid treatment in L6 myotubes. Intracellular lipids were assessed in L6 myotubes via targeted liquid chromatography tandem mass spectrometry during no fatty acid control (CON), palmitate (PALM, 500 *μ*mol/L), or oleate (OLEA, 500 *μ*mol/L) treated conditions. (A) Total intracellular diacylglycerol abundance, (B) 1,3‐diacylglycerol and (C) 1,2‐diacylglycerol species abundance. Diacylglycerol species abundance is presented as PALM and OLEA relative to CON, with the dashed line representing the mean value for CON. Intracellular lipid abundance was analyzed by one‐way ANOVA with Dunnett's posthoc analysis comparing fatty acid treatments to no fatty acid control. Lipid analysis reflects two independent experiments each consisting of *n* = 3–4 for a total sample size of *n* = 7 for all conditions. Data are presented as mean + SD. *P < 0.05 versus CON.

**Figure 3:**
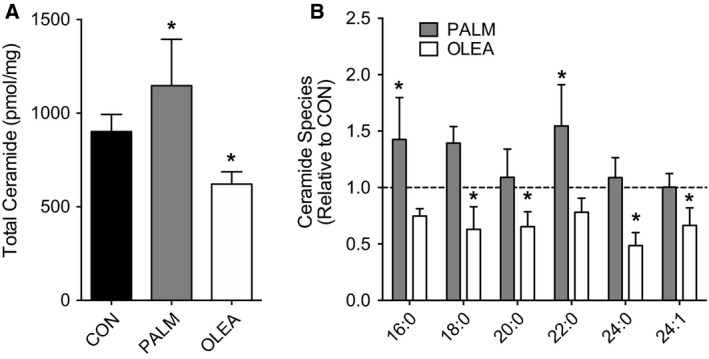
Ceramide abundance following overnight fatty acid treatment in L6 myotubes. Intracellular lipids were assessed in L6 myotubes via targeted liquid chromatography tandem mass spectrometry during no fatty acid control (CON), palmitate (PALM, 500 *μ*mol/L) or oleate (OLEA, 500 *μ*mol/L) treated conditions. (A) Total intracellular ceramide abundance and (B) ceramide species abundance. Ceramide species abundance is presented as PALM and OLEA relative to CON, with the dashed line representing the mean value for CON. Intracellular lipid abundance was analyzed by one‐way ANOVA with Dunnett's posthoc analysis comparing fatty acid treatments to no fatty acid control. Lipid analysis reflects two independent experiments each consisting of *n* = 3–4 for a total sample size of *n* = 7 for all conditions. Data are presented as mean + SD. *P < 0.05 versus CON.

We next determined the effect of overnight lipid treatments on accumulation of DAG and ceramides. Both total DAG and ceramide abundance were significantly increased following overnight incubation with palmitate compared with a no fatty acid control condition (Figs. 2A and 3A). Such accumulation of lipids was largely attributable to increased abundance of 1,2‐ and 1,3‐DAG containing C16:0 moieties (Fig. 2B and C), as well as increased C16:0, C18:0, and C22:0 ceramide abundance (Fig. 3B). Treating myotubes with oleate resulted in no change in total DAG abundance and reduced total ceramide abundance compared with the no fatty acid control condition (Figs. 2 and 3).

### Palmitate does not impair insulin‐stimulated Rac1‐GTP binding in L6 myotubes

**Figure 4:**
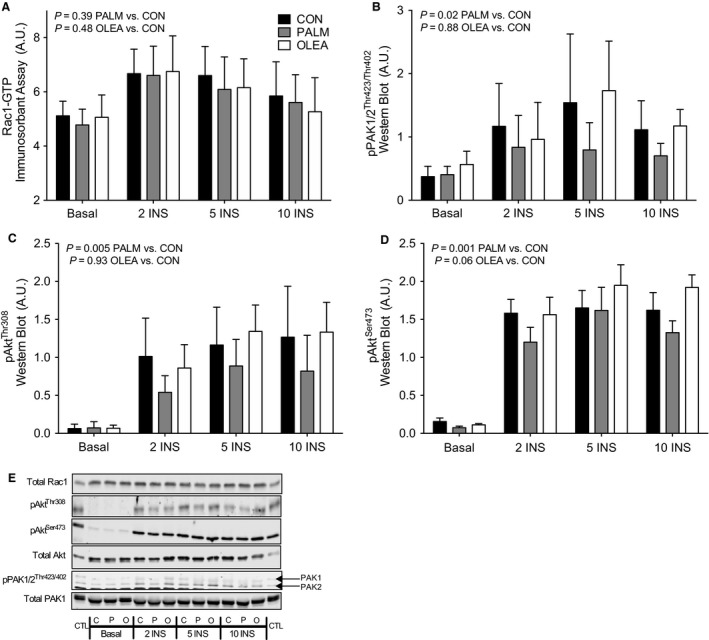
Insulin signaling following overnight fatty acid treatment in L6 myotubes. Insulin signaling in L6 myotubes following overnight treatment in no fatty acid control (CON), palmitate (PALM, 500 *μ*mol/L) or oleate (OLEA, 500 *μ*mol/L), under basal serum‐free or 100 nmol/L insulin for 2, 5 or 10 min. (A) Rac1‐GTP binding was assessed via immunosorbent assay. Western blotting was used to assess (B) phosphorylation of PAK 1 and PAK2 (pPAK1/2 Thr423/Thr402), and (C–D) phosphorylation of Akt at Thr308 (pAkt Thr308) and Ser473 (pAkt Ser473). (E) Representative western blot images. Insulin signaling outcomes were analyzed by two‐way *time* *×* *treatment* ANOVA. A main effect of insulin was evaluated by comparing 2, 5, and 10 min of insulin stimulation with basal using Dunnett's analysis. Main effects of fatty acid treatments were evaluated by comparing palmitate and oleate with the no fatty acid control condition using Dunnett's analysis. Total protein abundance of insulin signaling proteins was not affected by insulin or fatty acid treatments. Main effect *P*‐values for time and treatment, respectively, are provided for total Rac1 (*P* = 0.33, *P* = 0.31), total Akt, (*P* = 0.76, *P* = 0.17) and total PAK1, (*P* = 0.39, *P* = 0.95). Insulin signaling analysis reflects three independent experiments each consisting of *n* = 3 for a total sample size of *n* = 9 for all conditions. Data are presented as mean + SD.

We next determined insulin‐stimulated Rac1 activation following overnight fatty acid treatment. Despite robust DAG and ceramide accumulation during palmitate treatment, insulin‐stimulated Rac1‐GTP binding was not altered compared with no fatty acid control or oleate treatment (Fig. 4A). Palmitate treatment did, however, increase inhibitory phosphorylation of IRS1 at Ser1101 (*P *=* *0.02, PALM vs. CON; data not shown) and attenuate insulin‐stimulated phosphorylation of Akt at both Thr308 and Ser473 (Fig. 4C and D). In agreement with other models of lipid‐induced insulin resistance (Sylow et al. 2013), palmitate treatment also blunted insulin‐stimulated PAK phosphorylation compared with no fatty acid control (Fig. 4B). Overnight lipid treatment had no effect on total protein abundance of Rac1, Akt, or PAK1 (Fig. 4). Together these data indicate insulin‐stimulated Rac1‐GTP binding is not impaired by the accumulation of DAG and ceramides, yet PAK phosphorylation was attenuated during treatment with palmitate.

### Insulin‐stimulated GLUT4 translocation is blunted by palmitate in L6 myotubes

**Figure 5:**
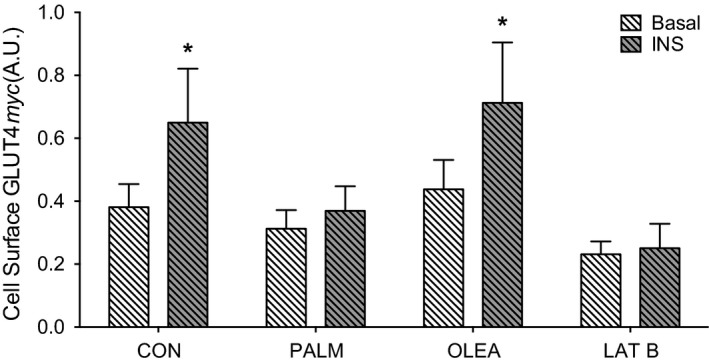
Insulin‐stimulated GLUT4 translocation following overnight fatty acid treatment in L6 myotubes. Glucose transporter protein 4 (GLUT4) translocation was assessed via cell surface detection of *myc* in L6 myotubes with stable expression of *myc*‐tagged GLUT4 following overnight treatment in no fatty acid control (CON), palmitate (PALM, 500 *μ*mol/L), oleate (OLEA, 500 *μ*mol/L) during basal serum‐free or 100 nmol/L insulin for 20 min. Latrunculin B (LAT B, 5 *μ*mol/L) was administered during the last hour of serum starvation in the LAT B condition. Cell‐surface GLUT4*myc* during basal and insulin‐stimulated (INS) conditions was analyzed via unpaired two‐tailed students *t*‐test. GLUT4 translocation analysis reflects three independent experiments each consisting of *n* = 3 for a total sample size of *n* = 9 for all conditions. Data are presented as mean + SD. *P < 0.05 versus Basal.

The significance of palmitate‐induced lipid accumulation and related impairments in insulin signaling was assessed via GLUT4 translocation. Insulin stimulation increased the content of GLUT4 detected at the cell surface during the no fatty acid control condition (*P *<* *0.001 vs. basal), with no adverse effect of oleate treatment (*P *<* *0.001 vs. basal, Fig. 5). Conversely, insulin‐stimulated GLUT4 translocation was blunted during treatment with either palmitate (*P *=* *0.10 vs. basal) or latrunculin B (*P *=* *0.70, Fig. 5), an inhibitor of actin polymerization (Spector et al. 1989). These data indicate that palmitate‐induced lipid accumulation and related impairments in insulin signaling effectively prevent insulin‐stimulated GLUT4 translocation in L6 myotubes and further demonstrate the need for a functional actin cytoskeleton during insulin‐stimulated GLUT4 translocation.

## Discussion

The purpose of this study was to identify the role for lipids as negative regulators of Rac1 in muscle cells. We hypothesized that palmitate‐induced accumulation of DAG and ceramides would impair insulin‐stimulated Rac1‐GTP binding. In contrast to our hypothesis, activation of Rac1 was not impaired by overnight palmitate treatment, in spite of significant accumulation of DAG and ceramides. Nevertheless, phosphorylation of PAK, a downstream target of Rac1 signaling, was impaired. Deleterious effects of palmitate were also observed for IRS1 and Akt, resulting in almost complete inhibition of insulin‐stimulated translocation of GLUT4. Together these findings suggest that lipid‐induced impairments in myocellular insulin action may be independent of defects in insulin‐stimulated Rac1‐GTP binding.

It is important to understand interactions between intramuscular lipid accumulation and impaired translocation of GLUT4. Rac1 has emerged as a critical regulatory point of skeletal muscle insulin signaling based on pharmacological inhibition studies and knockout models demonstrating impairment in insulin action (Sylow et al. 2013, 2014). Nevertheless, direct measures of Rac1 activation during models of lipid‐induced insulin resistance were needed. Here we report no impairment in Rac1‐GTP binding despite significant accumulation of DAG and ceramides, inhibitory signals for upstream regulators of Rac1 (e.g., IRS1), and attenuated activation of downstream targets (e.g., PAK). Our findings advance the field by demonstrating diminished activation of PAK and attenuated GLUT4 translocation can occur without impairment to Rac1‐GTP binding. These findings also show that Rac1‐GTP binding is preserved despite evidence for inhibition of proximal insulin signaling. The regulation of insulin‐stimulated Rac1‐GTP binding is not fully resolved but involves PI3K‐dependent activation of guanine nucleotide exchange factors (GEFs) (Das et al. 2000; Rosenfeldt et al. 2004; Tybulewicz 2005; Chiu et al. 2011). Additional evidence suggests that Rac1 can also undergo regulation that is PI3K‐independent (Lambert et al. 2002). How stimulation of Rac1‐GTP binding can be maintained during insulin resistance and if other impairment to Rac1 activation is present during insulin resistance, including altered localization, remain important questions of interest.

Our findings agree with previous reports demonstrating impaired phosphorylation of PAK in models of insulin resistance. Indeed, insulin‐stimulated phospho‐PAK was attenuated in obese humans and those with type 2 diabetes, high fat‐fed and ob/ob mice, and during acute infusion of lipids in otherwise lean, healthy adults (Sylow et al. 2013, 2014). While these findings implicated defects in Rac1 signaling, our current data suggest that such impairment in activation of PAK may be independent of dysregulated Rac1. This possibility is further supported by evidence that inducible knockout of Rac1 in skeletal muscle exacerbates high fat diet‐induced insulin resistance in mice (Raun et al. 2018). These findings indirectly suggest that Rac1 remains responsive to insulin in the high‐fat fed mice, otherwise knocking it out would presumably result in no further impairment in insulin action. Insulin‐stimulated Rac1‐GTP binding was not reported during conditions of insulin resistance in these previously published models (Sylow et al. 2013, 2014).

The activation of PAK is a multi‐step process that requires both GTP‐bound Rac1 to bind the autoregulatory region and phosphorylation at Thr423 in the kinase activation loop (Zenke et al. 1999). In the nonactivated state PAK exists as a homodimer, whereby binding of GTP‐bound Rac1 disrupts this dimerization causing conformational change to rearrange the kinase activation loop to a catalytically competent state (Lei et al. 2000). Previous evidence suggests that an exogenous kinase such as phosphoinositide‐dependent kinase 1 (PDK1) may be required for phosphorylation of PAK at Thr423, as autophosphorylation at this residue does not readily occur (King et al. 2000). In this study, phosphorylation of PDK1 at Ser241, which is essential for PDK1 activation (Casamayor et al. 1999), was not impaired during treatment with palmitate (data not shown). However, Akt phosphorylation at Thr308 was attenuated and is a known PDK1 phospho‐site (Sarbassov et al. 2005). Previous reports in C2C12 myotubes have demonstrated inhibitory phosphorylation of PDK1 during incubation with high concentrations of palmitate (Wang et al. 2009). These findings suggest impaired PDK1 activity may contribute to attenuated PAK phosphorylation during skeletal muscle insulin resistance.

The current findings did not support our hypothesis that accumulation of DAG and ceramides negatively regulates Rac1. This was unexpected given evidence from other model systems demonstrating negative regulation of Rac1‐GTP binding by DAG (Wang and Kazanietz 2006; Sosa et al. 2009). Some proteins important for regulation of Rac1 in these other cell types have low (or no) expression in skeletal muscle (e.g., *β*2‐chimaerin), which may explain this discrepancy. We also acknowledge that cultured muscle cells differ in protein expression and structure from fully developed skeletal muscle in vivo (Ravenscroft et al. 2007; Tondeleir et al. 2009). Nevertheless, a C2‐ceramide analog was previously shown to impair insulin‐stimulated Rac1‐GTP in L6‐GLUT4*myc* myotubes (JeBailey et al. 2007). The mechanism for this effect was unknown, but was not recapitulated in the current study during palmitate‐induced accumulation of C16:0, C18:0, and C22:0 ceramides. We used targeted lipidomics to identify molecular species involved with impairments to insulin signaling. The position and species of acyl chains affect signaling properties and thus regulatory functions of these lipids (Ritter et al. 2015). Further, many of the DAG and ceramides that accumulated during palmitate treatment have been linked to insulin resistance (Szendroedi et al. 2014; Chung et al. 2017). Our results cannot exclude the possibility that Rac1 may be negatively regulated by species of DAG and ceramides other than those which accumulated during palmitate treatment, but do agree with numerous reports showing that incubating muscle cells in oleate does not impair insulin action (Coll et al. 2008; Gao et al. 2009; Peng et al. 2011).

In conclusion, insulin‐stimulated Rac1‐GTP binding was not impaired in L6 myotubes during palmitate‐induced accumulation of DAG and ceramide. Nevertheless, PAK phosphorylation and GLUT4 translocation were attenuated during treatment with palmitate, suggesting that these impairments can occur independent of defects in insulin‐stimulated Rac1‐GTP binding. Mechanisms responsible for attenuated PAK phosphorylation remain unresolved, but may involve impaired activation of upstream kinases such as PDK1. Our findings further support the critical need for functional actin remodeling for skeletal muscle insulin action and the potential role for effectors of Rac1, such as PAK, in the development of insulin resistance.

## Conflict of Interest

The authors declare no conflict of interest.
